# Effect of Process Parameters on the Performance of Drop-On-Demand 3D Inkjet Printing: Geometrical-Based Modeling and Experimental Validation

**DOI:** 10.3390/polym14132557

**Published:** 2022-06-23

**Authors:** Ahmed Elkaseer, Stella Schneider, Yaqi Deng, Steffen G. Scholz

**Affiliations:** 1 Institute for Automation and Applied Informatics, Karlsruhe Institute of Technology, 76344 Eggenstein-Leopoldshafen, Germany; scst1085@h-ka.de (S.S.); ubvbe@student.kit.edu (Y.D.); steffen.scholz@kit.edu (S.G.S.); 2 Karlsruhe Nano Micro Facility, Karlsruhe Institute of Technology, Hermann-von-Helmholtz-Platz 1, 76344 Eggenstein-Leopoldshafen, Germany; 3 Faculty of Engineering, Port Said University, Port Fuad 42526, Egypt; 4 Future Manufacturing Research Institute, College of Engineering, Swansea University, Swansea SA1 8EN, UK

**Keywords:** 3D inkjet printing, geometrical-based simulation, TIFF resolution, coverage percentage, droplet diameter, droplets coalescence

## Abstract

As additive manufacturing has evolved, 3D inkjet printing (IJP) has become a promising alternative manufacturing method able to manufacture functional multi-material parts in a single process. However, issues with part quality in terms of dimensional errors and lack of precision still restrict its industrial and commercial applications. This study aims at improving the dimensional accuracy of 3D IJP parts by developing an optimization-oriented simulation tool of droplet behavior during the drop-on-demand 3D IJP process. The simulation approach takes into consideration the effect of droplet volume, droplet center-to-center distance, coverage percentage of jetted droplets, the contact angle of the ink on the solid substrate and coalescence performance of overlapping droplets, in addition to the number of printed layers. Following the development of the simulation tool using MATLAB, its feasibility was experimentally validated and the results showed a good agreement with a maximum deviation of 2.25% for horizontal features. In addition, the simulated horizontal features are compared with the results of “Inkraster” software, which also illustrates droplet behavior, however, only in 2D. For vertical features, a dial gauge indicator is used to measure the sample height, and the validation results show that the simulation tool can predicate the height of the sample with an average error of 10.89% for a large droplet diameter and 8.09% for a small diameter. The simulation results were found to be in a good agreement with the dimensions of the printed parts. The developed tool was then used to elucidate the effect of resolution of processed TIFF image and droplet diameter on the dimensional accuracy of 3D IJP parts.

## 1. Introduction

Additive manufacturing (AM), which in contrast to conventional manufacturing techniques constructs a part layer-by-layer, has undergone extensive development since its emergence in 1987 [1]. Different AM techniques have been developed and improved by individual researchers and industrial laboratories, each focused on different aspects such as materials, processes and/or applications [2]. The key advantages of AM (also known as 3D printing) are its limitless design freedom with regard to shape and materials [3], short lead times, the possibility to produce small batch sizes (even batches of just one) and customized parts [4]. At first, 3D printing was used mainly to produce prototypes during the development of new products. However, with improvements in the process and part quality, the potential to use it as part of manufacturing processes has grown, especially in the medical [5], construction [6], automotive, aerospace and electrical sectors [2,7].

Furthermore, the introduction of more and more materials which can be applied in 3D printing has brought up new fields of application [8]. Where at the beginning only some metal, polymer or resin materials were applicable, nowadays range of different metal and metal-based compounds as well as many polymers are available. However, in comparison to conventional manufacturing techniques, the amount of materials available for 3D printing is still limited [9]. Further challenges arise in the mechanical properties of 3D-printed parts due to the layer structure associated with ineffective or faulty bonding at the interlayer interfaces [10].

On the other hand, 3D printing offers the possibility of including several materials such as conductive [11], flexible, metal or ceramic materials or fibers [12] in a single component, during the same production process. This has also enabled the advent of functional printing with which shape can be generated and functionality can be integrated during the printing process. The unrivaled development rate of functional 3D printing is mainly owing to the fact that no retooling is necessary, which means parts can be optimized regarding their function instead of having to modify the manufacturing process for each production batch [13].

As of today, AM includes seven main technologies which can be divided into three subcategories, including material extrusion, powder bed fusion and material jetting [14,15]. A subsection of the latter category is inkjet printing (IJP), an advanced technology based on conventional inkjet printing which is found in many households and offices. Three-dimensional IJP is also called material jetting or polymer jetting and is part of the liquid-based layer additive manufacturing group [14]. In the electrical sector, 2D/3D IJP is widely applied to manufacture sensors and circuit boards; however, it is also a promising technology for other sectors, such as the health or energy harvesting sectors and monitoring applications, due to its high resolution and resulting high scalability. Furthermore, in contrast to the commonly found powder bed 3D printing technologies, the handling of multiple different materials is much more convenient, and hence the creation of fully functional 2D and 3D multi-material parts is potentially achievable [16,17].

To be able to use 3D IJP for industrial applications, it is crucial for the printed parts to be accurate and within agreed production tolerances. However, dimensional accuracy as well as other quality characteristics, such as surface texture, are still an issue when it comes to 3D printing. Unlike extrusion-based processes, such as in Fused Filament Fabrication (FFF), where the quality of printed parts mostly depends on the material and the input parameters of the printer, in 3D IJP, the CAD slices of the part in the form of TIFF files have a significant effect on the outcome. An important characteristic of these TIFF files is their resolution, which controls the center-to-center distances of the dispensed droplets and helps to modify and optimize the process outcome. In particular, a TIFF file with a higher resolution leads to a smaller center-to-center distance of dispensed droplets and thus less variation between segments printed by different nozzles of the printhead. This also improves the smoothness and clearness of the outlines of the part. Nevertheless, it is a time-consuming process and increases the amount of material dispensed, thereby to some extent increasing the final dimensional error of the printed features when compared with the required CAD design. The coverage percentage is another significant feature of the input TIFF file that can be optimized via applying a dithering technique to remove some of the active pixels in the TIFF image in order to control the final output of the inkjet process. This can maintain the positive effect of the high resolution and, therefore, result in high accuracy with less variation between segments printed by different nozzles. However, at the same time, it reduces the amount of material dispensed, and hence might produce porous parts if it is incorrectly applied. This could be compensated by using larger drops, although this might lead again to an increasing dimensional error.

In the published literature, a number of studies can be found on optimizing and simulating 3D IJP. Tourloukis et al. [18,19] developed a predictive model based on artificial neural networks to reduce the dimensional errors in the height of printed parts. Several data sets containing information about the layer height and droplet volume were fed into the algorithm as training materials. Overall, they found the algorithm to be efficient at decreasing significant deviations in general, but also for higher step ahead predictions.

Xu et al. [20] studied the correlation between the ink drop size and the covered area and created a prediction method stating that a variation of one percent of the covered area corresponded to a drop diameter change of 1 μm.

Lu et al. [21] and Guo et al. [22,23,24] developed, in several consecutive studies, a layer-by-layer closed-loop feedback control algorithm for 3D IJP. By observing and measuring the height within the printing process using a height measuring sensor, the algorithm detects deviations in layer height and profile during printing and sends signals to the printer, which is then able to correct errors immediately.

Based on this work, Inyang-Udoh et al. [25,26] improved the computation time of the model and extended the algorithm by a recurrent neural network to be able to also consider different drop shapes and non-linear material flow.

Salcedo et al. [27] established a model to predict the deformation and stress concentration of parts printed with multiple materials, such as flexible and rigid ones, based on finite element analysis. The model was validated with physical tensile tests, and it was shown that it was capable to determine the maximum strain of the material with a 5% error.

Zhou et al. [28] established an approach to determine the printability of various materials and composites by introducing a one-stop liquid handling station, which performed measurements such as viscosity and surface tension measurements. To verify the new approach, they compared the results obtained with the high-throughput scanning approach with results from conventional assessment methods and found them to be matching. Moreover, the ideal processing temperature was identified by the developed tool.

Wu et al. [29] numerically and geometrically examined and simulated the formation process of ink drops. Therefore, the parameters of surface tension, frequency and entrance driving speed were closely looked at. One optimization criterion was, among others, a reduction in satellite drops. This was achieved by applying lower frequencies and entrance driving speed, as well as higher surface tension.

From the literature review, it can be noticed that there are a number of recent research studies in the field of 3D IJP and investigations are directed in different directions, such as the usability of materials, ink characteristics and part quality, e.g., dimensional accuracy or mechanical properties, as well as optimal process parameters. Nevertheless, it is apparent that there is still a gap regarding the possibility to use experimentally validated simulations to quantify the effect of governing parameters on the performance of the printing process for the best possible outcome before the printing process is started. Especially, TIFF properties are governing input factors on the process outcome, are easily modifiable, and comprise the resolution (dpi) as well as the pixel coverage percentage, with both having an effect on the deposition of the ink drops. So, the overall goal of this project is to develop a simulation tool using MATLAB to study the effect of the combination of resolution and coverage percentage of the TIFF files in addition to the drop diameter on the inkjet printing performance, and hence to identify a working window for the abovementioned processing parameters, to achieve a higher dimensional accuracy of 3D inkjet-printed parts, and to give some recommendations on how to achieve fully functional and solid parts.

Proceeding from this introduction, this paper is organized as follows: First the theoretical background of 3D IJP and droplets is explained. Next, the development of the simulation tool, the validation of its feasibility and the results are presented and discussed. Finally, conclusions are drawn, and possible future work is proposed.

## 2. Theoretical Background

### 2.1. The 3D Inkjet Printing Process

**Figure 1:**
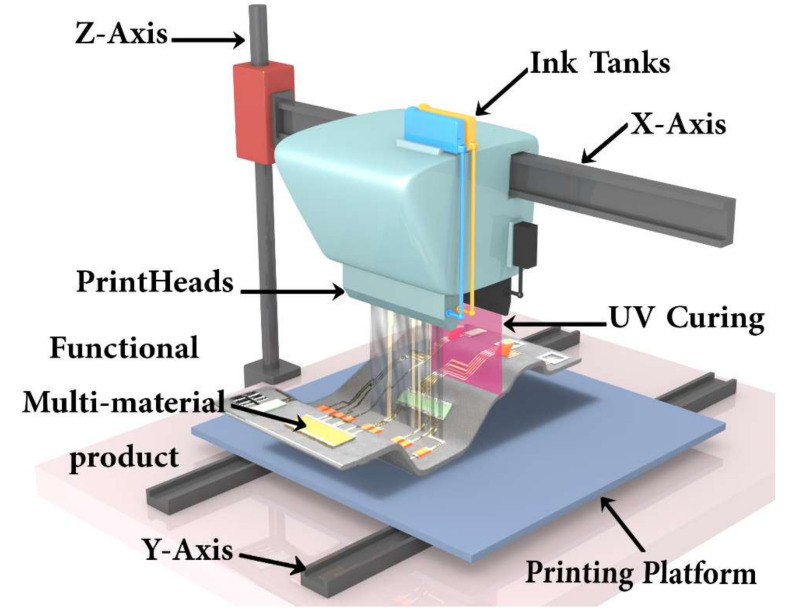
Schematic of the 3D inkjet printing process.

Three-dimensional inkjet printing (IJP) is a material jetting technique in additive manufacturing (AM) [14]. AM includes different approaches to create components layer-by-layer. Apart from jetting processes, where liquid photopolymers or powder particles are solidified/bonded through an UV light source or a bonding agent, laser-based or extrusion-based methods are also commonly found in 3D printing. The former melts the powdered material layer-wise by a laser beam, while the latter heats up a filament-formed polymer, which is then extruded through a nozzle and deposited in a predefined path onto a building platform [30,31]. In the IJP process, a liquid photopolymer is jetted onto a substrate and immediately cured by an ultraviolet (UV) light source. Then, the platform with the substrate moves down and a new layer of drops is applied, cured, and connected to the already built part. In order to create overhang or a new layer after a hollow form, a support material is necessary (see Figure 1). Hence, each printer contains at least two different print heads; each is equipped with a varying number, e.g., 16 to 1024, of linearly arranged nozzles [32].

The IJP process can be divided into two working principles regarding the ejection mechanism of the droplets. First is the continuous inkjet printing (CIP) mode where a continuous stream is ejected through the nozzle which then separates into droplets due to Rayleigh instability. To place the droplets precisely on the substrate, they are charged by passing an electrostatic field created by a charging electrode and then directed by a deflection electrode. Unused droplets are caught by a gutter and can be reused directly. In contrast, using the second mode, drop-on-demand (DOD), it is possible to eject the droplets when needed—controlled by digital signals—and hence place them at an exact spot on the substrate by either moving the substrate or the inkjet printing head [33].

To eject the droplets on-demand from the nozzle, a thermal or piezoelectric actuator controlled by an actuation pulse voltage can be used. The former method makes use of a heater which could cause the liquid ink to form a bubble. In case of the latter, the voltage causes a deformation of the piezoelectric actuator. Hence, in both methods, a reduction in chamber volume occurs so that the ink is pressed together and forced to eject a drop [16,28,34]. To emit the drop, a voltage is applied for a short period before it is reduced to a negative value in order to decline any vibrations left inside the liquid [35]. This method is more common nowadays since it does not require heating up the ink, which might damage it, and which limits the amount of useable ink. Additionally, it is possible to define the droplet characteristics beforehand in more detail, as the piezoelectric actuator and its controlling pulse can be finetuned better than the thermal jetting mechanism [12,34].

### 2.2. Physics of Droplets in Inkjet Printing

Two crucial properties of fluids, i.e., inks, for IJP are surface tension γ and viscosity η. The surface tension is the dominant force responsible for the drop adopting a spherical shape if it is in contact with only the ambient air and is not significantly influenced by other forces, such as gravitational or aerodynamic forces. The viscosity can be described as the resistance of a liquid to shear deformation or flow. Due to a cohesive intermolecular force, friction can be said to occur between layers of the fluid moving relative to each other [36].

In order to determine, whether a fluid is properly printable, meaning it is not too fluid or too viscous, some dimensionless groups of physical constants are introduced [34,36]. The Reynolds number *Re* (Equation (1)) describes the ratio between inertial and viscous forces in a moving fluid, where ρ is the density, *V* the velocity and *d_n_* the nozzle diameter.

(1) $$Re=\frac{\rho \;V\;d_{n}}{\eta }$$

Whereas the Weber number *We* (Equation (2)) illustrates the ratio between inertia and surface tension.

(2) $$We=\frac{\rho \;V^{2}d_{n}}{\gamma }$$

With these two numbers, the Ohnesorge Number *Oh* (Equation (3)) can be calculated and the influence of the Velocity *V* removed, so that it only reflects the physical properties of the liquid and the size scale of the droplet.

(3) $$Oh=\frac{\sqrt{We}}{Re}=\frac{\eta }{\sqrt{\gamma \;\rho \;d_{n}}}$$

When a droplet impacts onto a substrate it will oscillate before it forms its capillary-driven equilibrium shape, which is a spherical cap [34]. The diameter of this spherical cap can be determined from the initial droplet diameter *d*_0_ and the equilibrium contact angle *θ_eqm_* by Equation (4) [37].

(4) $$d_{eqm}=d_{0}*\sqrt[{3}]{\frac{8}{\tan\frac{\theta_{eqm}}{2}*\left(3+\tan^{2}\frac{\theta_{eqm}}{2}\right)}}$$

A major factor in the equations representing a fluid on a solid substrate is the contact angle, which depends on the surface tension between the liquid and the solid, as found from the Young equation [38].

When two drops coalesce, they first connect over a meniscus bridge which then expands further until the drops fully merge into one hemispherical cap, provided that the drop spacing, i.e., the center-to-center distance, is smaller than the drop diameter [39,40].

This process is also applicable to the formation of a line of drops. If several drops are deposited onto the substrate linearly, they will form a liquid line which has a hemispherical cross-section. The width *w* of this line will depend on the initial droplet diameter *d*_0_, the droplet spacing/center-to-center distance *p*, and the contact angle *θ_eqm_* (see Equation (5)) [37].

(5) $$w=\sqrt{\frac{2\pi d_{0}{}^{3}}{3p\left(\frac{\theta_{eqm}}{\sin^{2}\theta_{eqm}}-\frac{\cos\theta_{eqm}}{\sin\theta_{eqm}}\right)}}$$

**Figure 2:**
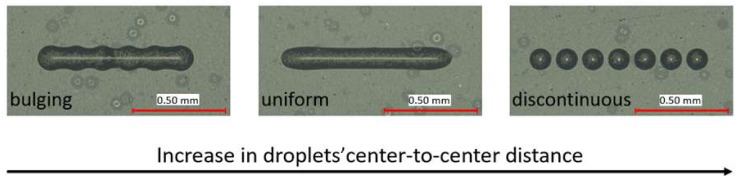
Impact of droplets’ center-to-center distance on line formation.

Nevertheless, the stability of the line depends, in part, on the droplet center-to-center distance. If the droplets are deposited too far from or too close to each other, either bulges occur or the line becomes discontinuous (see Figure 2).

This bulging of the line and the following instability were closer examined by Duinevelt [41]. In his study, he investigated conditions for unstable lines and how to describe their shape and geometry in terms of mathematical models.

**Figure 3:**
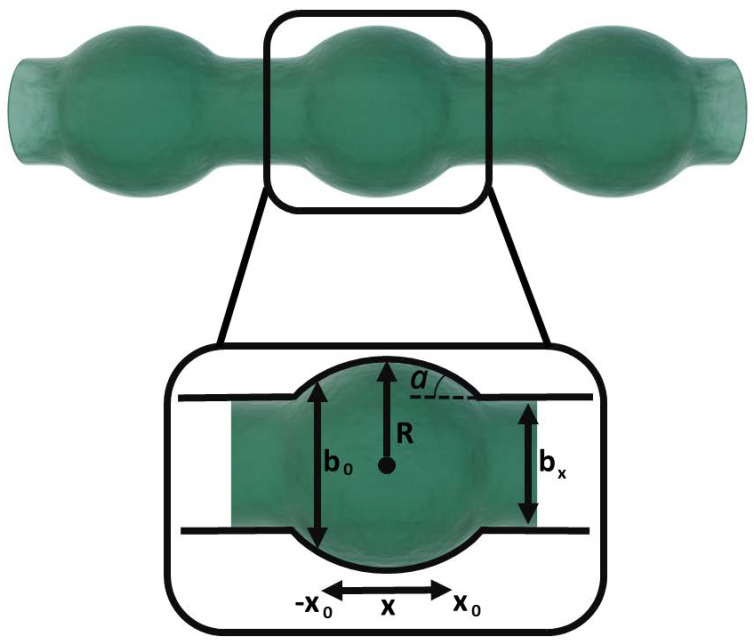
A bulge in the middle of a stable line with a constant width b0 (inspired from [41]).

In order to describe the geometry of a bulge, first its shape regarding the curvature in line with the ridge is examined, meaning the top view onto the line (see Figure 3). The bulge can be seen as a trimmed circle with radius *R*, which intersects with the ridge of the line at an angle *α*. The outline of the circle can be defined by Equations (6) and (7).

(6) $$b\left(x\right)=b_{0}+2\left(\sqrt{R^{2}-x^{2}}-R\cos\alpha \right),\;\;\;\;\;-x_{0}\leq x\leq x_{0}$$

(7) $$x_{0}=R\sin\alpha$$

**Figure 4:**
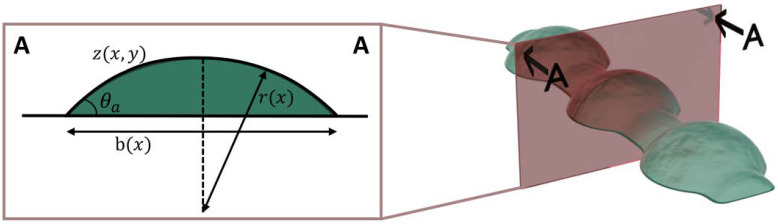
Cross-section of a bulge (inspired from [41]).

To describe the surface of the bulge, as well as any surface of an ideal bead, Equation (8) can be used. For the bulge, *r*(*x*) is defined in Equation (9), and hence for an ideal bead *b*(*x*) *= w* (see Figure 4).

(8) $$z\left(x,y\right)=\sqrt{r^{2}\left(x\right)-y^{2}}-r\left(x\right)\cos\theta_{a}$$

(9) $$r\left(x\right)=\frac{b\left(x\right)}{2\sin\theta_{a}}$$

**Figure 5:**
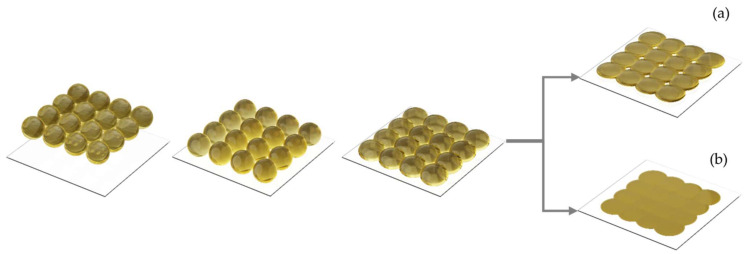
Illustration coalescence sequence of an array of drops leading to (**a**) with holes and a planar surface, and (**b**) without holes.

When placing several lines of drops next to each other, the drops do not form a line, but, if the droplet center-to-center distance is too wide then this surface will feature some holes (see Figure 5a). However, if the drop spacing is right, they will form an array of drops with a plane surface (Figure 5b) [42].

### 2.3. Tagged Image File Format

The Tagged Image File Format (TIFF) was introduced in 1986 by the companies Microsoft and Aldus. TIFFs handle different color depths, e.g., bilevel, greyscale or Red–Green–Blue (RGB). Hence, they are compatible with many different image processing devices such as printers, monitors or scanners. Moreover, the resolution—and resulting image size of the TIFF—is freely tunable. The image data are stored in so-called “tags”, thus the name Tagged Image File, preceded by a file header. Due to a wide range of different tags and many tags within one image data set, the file size of TIFFs is high [43,44].

Since, in this work, only bilevel TIFFs are of relevance, in the following these are described in detail. Bilevel images only contain two colors, in this case black and white, which are identified by the numbers zero and one. Depending on the photometric interpretation, either white or black is displayed as zero. One of the most crucial properties of a TIFF is the length and width of its array of pixels, i.e., the number of rows and columns. When this property is combined with the next important characteristic, namely the resolution, the size of the picture can be calculated. The resolution states the number of pixels, also called dots, per unit, most commonly referred to as dots per inch (dpi). However, centimeters are also a possible unit. It should be noted that different resolutions for the width and length are possible, referred to as X-resolution and Y-resolution [44].

## 3. Materials and Methods

### 3.1. Development of the Simulation Tool

**Figure 6:**
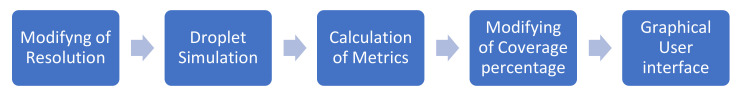
Main components of the developed simulation tool.

In this section, the development of the simulation tool with MATLAB 2020b is presented and the most important steps and functions (see Figure 6) are explained in detail.

In a first step, the binary code of the TIFF was stored in a matrix where each binary value (0 or 1) occupied one matrix element. Next, a short algorithm checked the value of each matrix element and, if 0 was found, the respective row and column numbers were saved in another matrix corresponding to the coordinate on the substrate (column number|row number) of this drop.

The main purpose of the simulation tool is to plot the drops on the substrate. Depending on the center-to-center distance between the ejected droplets, they either connect with the neighboring droplets or they do not connect and spread into a spherical cap. In reality, the droplets would coalesce with their neighbors in the x- and y-directions and form a shape with a plane top surface (as described further in Figure 5). To simplify the geometrical model proposed in this study, the approach for the simulation was implemented as follows. In a first step, depending on the distance, all droplet lines and single drops in the x-direction were plotted, ignoring the distance of the droplets in the y-direction. Then, in a second step, the distances in the x-direction were ignored and only the lines and single drops in the y-direction were plotted into the same figure and coordinate system. The final planar simulation involved a merge between the simulation results of both steps previously implemented in the x- and y-directions individually.

In the following, the approach to plot and model the drops in the x-direction is explained in detail, and the approach for the y-direction is the same.

**Figure 7:**
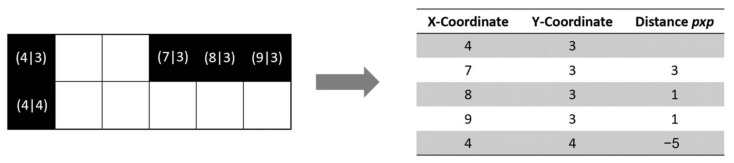
Example of extraction of drop coordinates and drop spacing depending on their position.

So, after extracting the positions of all black pixels from the TIFF data, the distance between the x-coordinate of the current droplet center and its previous location was calculated and saved in a new column of the matrix already containing the coordinates (see Figure 7).

Then, this value was multiplied with the pixel size in the x-direction to obtain the actual droplets’ center-to-center distances.

**Figure 8:**
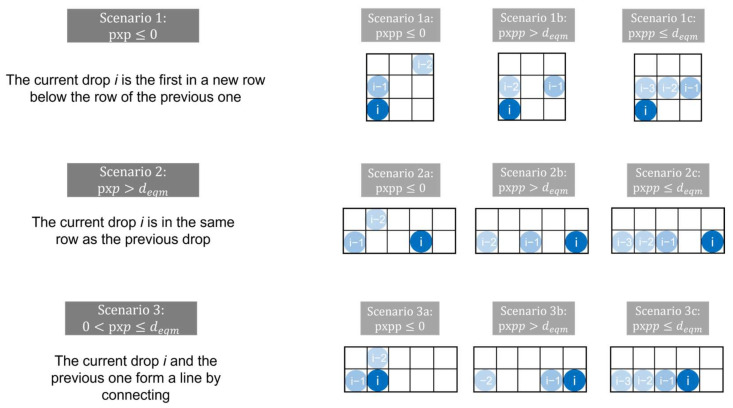
Possible droplet formations depending on the distance to the previous droplets.

Next, using a “for loop”, for each droplet starting from the second, this distance (in the following called *pxp*) was checked and, depending on its value, the next step is initiated. If *pxp* ≤ 0 or *pxp* > *deqm* then the droplet line was interrupted or a single droplet was deposited. In the first case, the droplet was deposited in a new row, since a negative distance indicated that the previous droplet was to the left of the current droplet. In the second case, the distance between the droplets was too wide so they could not connect. If this happened, either a droplet line ended with the previous droplet or a single droplet was plotted depending on the distance between the previous and the pre-previous droplet (*pxpp*). All the different scenarios are shown in Figure 8. The three different possibilities in Scenario 3 are only relevant for the last deposited drop. Else, if a distance meeting the condition 0 < *pxp* < *d_eqm_* was detected, nothing happened and the distance of the next drop was investigated, since in this case the droplet line would continue with the current drop.

For Scenarios 1a, 1b, 2a and 2b, the MATLAB code is the same, since for each scenario the previous drop is a single drop not connecting to the others, only differing in its position. However, the position of each drop is set after plotting the previous drop or line, and therefore it is not relevant where exactly the previous single drop is in relation to the current one.

After determining the scenario, either the “plotDrop” or “plotLine” functions were called.

To plot a single drop, the equations from Stringer et al. [35] and Duineveld [40], which were presented earlier, were modified and combined, because it is not possible to just plot the upper half of a sphere due to the varying contact angle.

First, the diameter of the droplet at the equilibrium was used to define the meshgrid—meaning two arrays forming a grid—containing the datapoints by which the surface would be shaped later. The step width of these datapoints, and hence the precision of the plotted mesh, was set by a variable, which should not be too low, because in this case the meshgrid would not be smooth, but also should not be too high, since then the number of datapoints rises and the process takes too long and requires too much data storage. Next, the base width *z*(*x*) was calculated using Equation (7), where *R* = *d_eqm_*/2, *α* = 0 and *b_0_* = *d_eqm_*, in order to be able to apply Equations (9) and (10), where *z*(*x*) serves as *b*(*x*) in Equation (9).

After the droplet surface shape was defined, the meshgrid was moved to the right position and, finally, the droplet was plotted based on the meshgrid and the calculated height (Z1).

**Figure 9:**
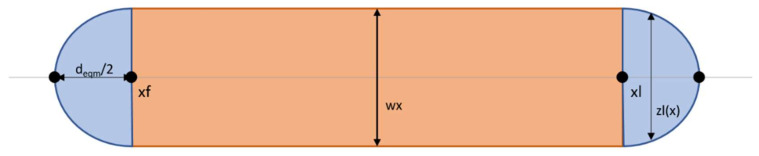
Base shape of a modelled drop line.

In comparison, when using the function “plotLine”, in the first step the last x-coordinate of the line (*xl*) is set. In the following step, the width of the line *w_x_* is calculated with Equation (6), where *d*_0_ = *d*, the initial droplet diameter, and *p* = *pxpp* since the line ends with the previous drop and therefore the distance between the previous and pre-previous droplet is determining the line width. Then, a meshgrid is again defined, beginning in the x-direction at the first droplet and ending at the last droplet, presuming that the droplets spread to an equilibrium shape before connecting. Regarding the y-direction, the meshgrid is circumscribed by *w_x_*. In order to be able to model the line correctly, it is divided into three different parts (see Figure 9).

For the base of the two outer parts (displayed in blue) Equation (6) was used again, with the difference that now *b_0_* = *w_x_* and the limitation *x* ≤ *xf* and *x* ≥ *xl* for the other half. The base of the middle part does not need to be defined as it is rectangular and does not change its width *w_x_.* Once more, Equations (8) and (9) were used to model the surface with

$$b\left(x\right)=\left\{\begin{array}{c} zf\left(x\right),\;x\leq xf\\ w_{x},\;xf<x<xl\\ zl\left(x\right),\;x\geq xl \end{array}\right.$$

Since the meshgrid is already at the right *x*-coordinate, it only needs to be moved to the right *y*-coordinate before the surface is plotted.

If the last ejected drop is the ending of a droplet line, then *pxp* is used to calculate the width *w_x_*.

**Figure 10:**
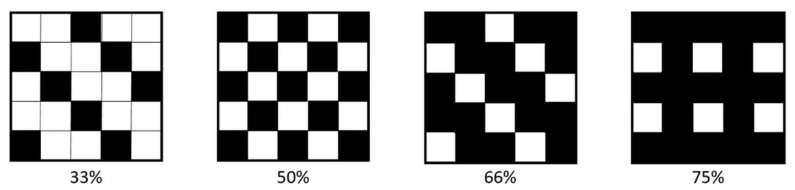
Selectable coverage percentages and their patterns.

The developed simulation tool is equipped with a dithering algorithm that can be utilized to modify the coverage percentage of the pixels of the TIFF data, according to the selected pattern (see Figure 10). The lower the coverage percentage, the less material is dispensed; however, depending on the resolution and the droplet diameter, the drops might not connect if the coverage percentage is too low.

Initially, the position of the first encountered black pixel in the TIFF was determined and saved. Next, looking at the process for a coverage percentage of 50%, by using the modulo operator (here: mod 2) on the coordinates of that pixel, it was possible to identify every second pixel based on the remainder (0 or 1) and set its value to 1 (white). Additionally, to create the checkerboard pattern, it was necessary to shift the “removed” pixel in every second row; therefore, the modulo operator was also used to change the columns (even/uneven) after each row in which the pixels were removed.

For the other coverage percentages, the procedure was adapted. For a coverage percentage of 33% and 66%, the above-presented algorithm was reused with the difference that the row and column indices were divided by three, and because of that three different remainders (0, 1, 2) resulted. For a coverage percentage of 75%, the above-presented algorithm was used with the difference that it was only applied to every second row without a shift over the rows.

### 3.2. Workflow of the Simulation Tool

In this section, the general functioning of the developed simulation tool is explained by providing a flow chart as well as simulation results.

**Figure 11:**
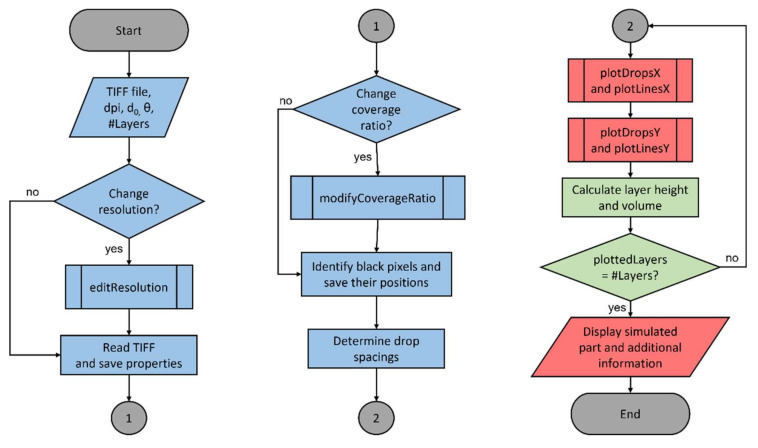
Flowchart of the developed simulation tool.

To obtain a general understanding of how the tool works and which steps are necessary, the functioning of the simulation tool is displayed in Figure 11 with a flowchart.

The functioning of the tool can generally be divided into three main groups: data preparation (blue), plotting and displaying (red), and processing the generated data (green).

After the necessary input parameters are inserted through the GUI, in a first step, the resolution of the TIFF is modified to the desired one. Next, the TIFF data are read into the program and properties such as the new resolution and the pixel bitmap are saved. If a lower coverage percentage is requested the TIFF pixel bitmap is modified with the respective coverage percentage pattern. Then, all the black pixels, which will later be covered with a drop, are identified and their positions saved. Using these positions, the distance between neighboring black pixels, and hence the droplet center-to-center distance, is determined and also saved. Depending on the droplet center-to-center distance, first, all single drops or drop lines in the X-direction are plotted, followed by the ones in the Y-direction. To be able to add further layers, the layer height needs to be calculated and the overall drop volume ascertained. If the number of layers is not reached yet, the tool goes back to plotting the drops based on their drop spacing for the next layer. When all layers are plotted, the final result is displayed and some output variables are given, such as the height of a single layer.

### 3.3. Validation

To validate the results produced by the simulation tool, two different approaches were chosen. The first approach was to simulate the printed parts with the same input parameters and conditions and to compare the simulation results with the measurements of the printed part.

The second approach was to use the software “Inkraster” and “TIFF2Droplet” by “Artworks” to simulate the printing process and to compare the results with those produced by the proposed simulation tool in this research study. With this software, “Inkraster” and “TIFF2Droplet”, similar to the tool developed in this work, the placement of the drops according to the pixels is simulated, but only two-dimensionally.

The material jetted for the printing validation tests was an EPJ-02 ink build material from BASF 3D Printing Solutions GmbH, Germany. EPJ-02 ink is a highly viscous UV-curable multipurpose material with balanced mechanical performance.

**Figure 12:**
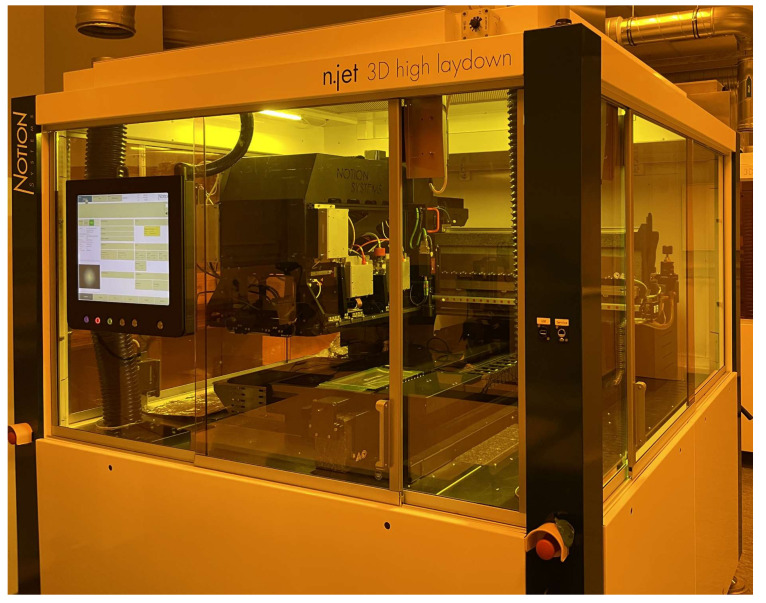
Notion Systems high laydown printer.

**Figure 13:**
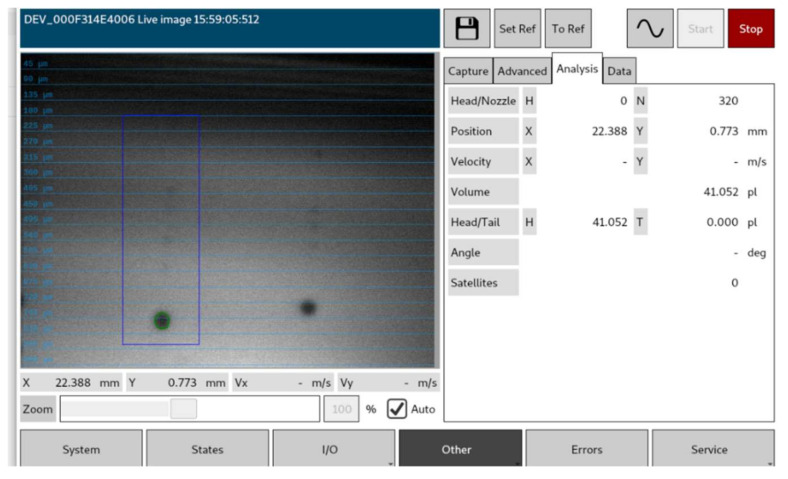
A dropwatcher of the Notion Systems machine characterizing in-flight droplets.

**Figure 14:**
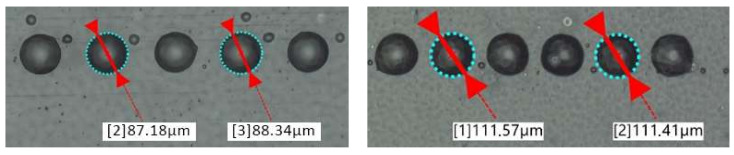
Characterization of droplets laying on the substrate.

The experimental printing trials were conducted on Notion Systems’ high laydown machine, Figure 12, equipped with two Midas 950 systems to recirculate inks and two Xaar 1003 printheads of 1000 nozzles and one Konica Minolta KM1024i printhead of 1024 nozzles. The Notion Systems machine enables a build volume of 156 × 200 × 80 mm (XYZ) and an accuracy of 1 µm in the X and Y axes and 3 µm in the Z axis. The machine is also equipped with a dropwatcher to characterize the droplet generation in flight (see Figure 13). In these experimental trials, the greyscale was varied in order to diverge the droplet diameter (*d*_0_) between 41.2 μm and 31.9 μm. The droplets laying on the substrate were also characterized using an optical microscope and the diameter of the spherical cap of the droplets in the equilibrium was also characterized and found to vary between ~88 and 111 µm (see Figure 14) and thus the contact angle (*θ_eqm_*) was determined to be 15° ± 0.5°. Finally, a dial gauge indicator with a measuring sensitivity of 0.5 µm was used for the vertical feature (printed layer height).

#### 3.3.1. Validation with 3D-Printed Parts for Horizontal Features

To validate the results produced by the simulation tool for horizontal features, the results were compared with a 3D-printed “dogbone” part by inserting the same parameters which were applied to the parts into the simulation tool. The parameters used were as follows.

- X-resolution dpi_x_: 360 dpi;
- Y-resolution dpi_y_: 360.2837 dpi;
- Number of layers: 100;
- Coverage percentage: 100%.

**Figure 15:**
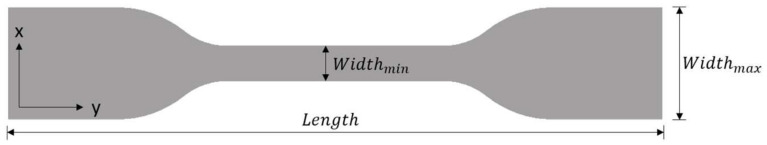
Ascertained dimensions for the dimensional error.

The dimensions of the printed dogbone were ascertained as shown in Figure 15 using a digital caliper at three printed parts.

#### 3.3.2. Validation with 3D-Printed Parts for Vertical Features

**Figure 16:**
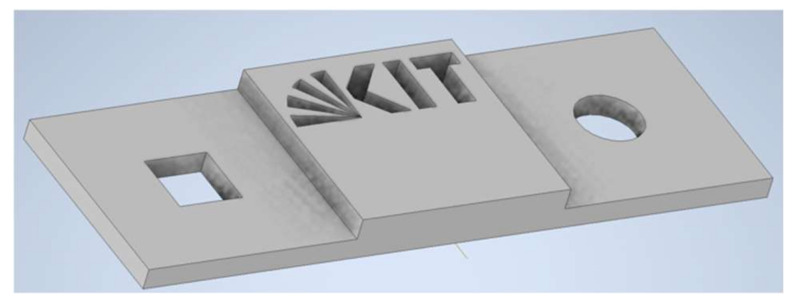
“KIT logo” sample for the validation of vertical-printed features.

Regarding the vertical features of the printed parts, two sets of samples with different combinations of resolution, droplet diameters and coverage ratios were printed. Due to the fact that the shrinkage after curing was not considered by the simulation tool, this led to an unprecise prediction of the samples’ heights. To optimize the results, height-correcting factors were introduced into the simulation which were extracted from the first set of samples. After introducing height-correcting factors, a second set of samples (“KIT logo” sample) was printed and simulated. The rectangular base had a size of 30 mm×10 mm and a 10 mm×10 mm square was positioned on top of the base. Both elements were printed with 10 layers and 5 layers, respectively (Figure 16). After introducing height-correcting factors, a second set of samples (“KIT logo” sample) was printed and simulated. The rectangular base had a size of 30 mm×10 mm and a 10 mm×10 mm square was positioned on top of the base. Both elements were printed with 10 layers and 5 layers, respectively (Figure 16).

The input parameters for the process simulation were:

- X-resolution dpi_x_: 360 dpi or 720 dpi or 1080 dpi;
- Y-resolution dpi_y_: 360 dpi or 715 dpi or 1080 dpi;
- Number of layers: 10 base layers and 5 top layers;
- Coverage percentage: 100% or 75%.

#### 3.3.3. Validation Using the “Inkraster” and “TIFF2Droplet” Software

**Figure 17:**
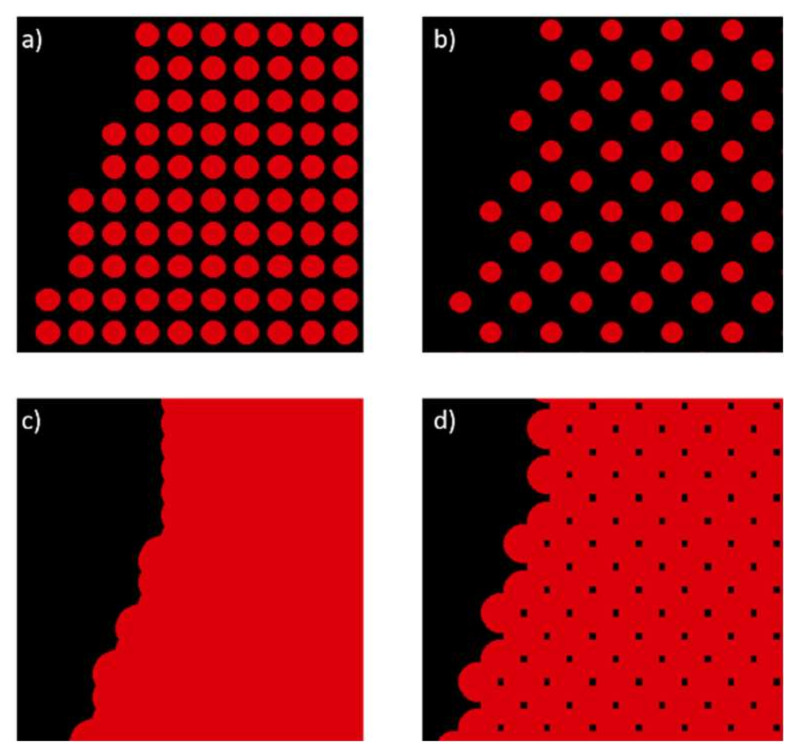
Output generated with “Inkraster” software: (**a**) drops do not connect, 100% coverage percentage; (**b**) drops do not connect, 50% coverage percentage; (**c**) drops connect, 100% coverage percentage; (**d**) drops connect, 50% coverage percentage.

To validate the other input parameter combinations, “Inkraster” and “TIFF2Droplet” software were used. Similar to the tool developed in this work, these software functions place a drop at each “black” pixel and simulate the model. However, the drops are not displayed three-dimensionally and the spreading of the drops on the substrate and the resulting widening of the part are not considered. Therefore, as the input value for the droplet diameter, the diameter at equilibrium *d_eqm_* was used, allowing a better comparability between the two tools. However, the coalescence of overlapping drops was not taken into account since only circles with the input diameter illustrating the droplets were allocated to each black pixel (see Figure 17).

Similarly to the developed simulation tool, a coverage percentage can be entered which results in the same patterns for a percentage of 50% and 75%, as shown above in 0, yet it is not possible to enter different resolutions regarding the x- and y-directions.

It should be noted that the main field of application of “Inkraster” is not 3D Inkjet printing, but rather the 2D Inkjet printing of printed circuit boards using chemical inks. Hence, it is mostly used to investigate the drop raster and to optimize the drop placement to avoid inaccuracies.

## 4. Results

**Figure 18:**
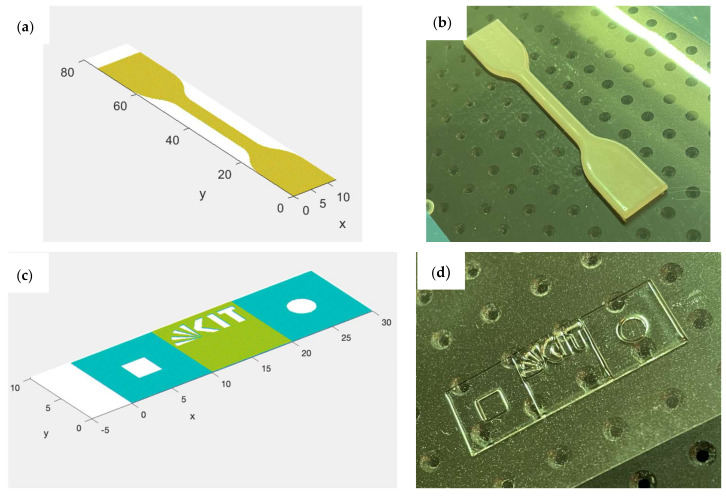
Experimentally validated samples, (**a**) a simulated dogbone sample (**b**) a printed dogbone part, (**c**) a simulated “KIT logo” sample and (**d**) a 3D-printed “KIT logo” part.

In Figure 18, examples of a simulated dogbone layer and “KIT logo” sample are visualized against the 3D-printed parts. On the left-hand side (a) the overall result of the simulated dogbone (but with limited number of layers, as simulating 100 layers consumes time and computational power and only horizontal features are examined in this trial) can be seen, while the real 3D-printed dogbone is illustrated in (b). Similarly, the simulated “KIT logo” sample is presented in (c), and (d) shows the printed “KIT logo” part under the same printing conditions.

### Quantitative Assessment of the Results

For the horizontal parameters, only one set of process parameters was validated with the printed dogbone structure. In addition, as formerly stated, the software “Inkraster” was used to simulate the horizontal dimensions of the printed dogbone as well. It has to be taken into account that the resolution in the y-direction could not vary from the one in the x-direction (360 dpi), which is why the simulated result for the length by “Inkraster” might be less accurate than the ones for the width.

**Figure 19:**
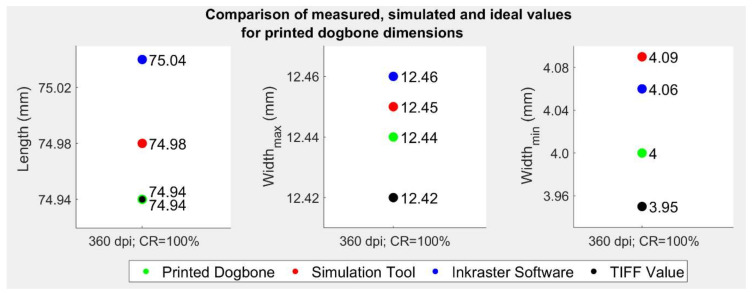
Results of the validation using printed dogbone parts (initial drop diameter = d_0_; contact angle θ; 360 dpi (X): 360.2837 dpi (Y); Coverage Percentage = CR).

In Figure 19, the ascertained metrics from the printed parts, the simulation tool and the software “Inkraster” are displayed, as well as the ideal metric value according to the TIFF.

It can be seen that the values are quite close to each other and only differ by several microns. Moreover, the values simulated by the developed tool and “Inkraster” are constantly higher than the ones measured at the printed parts, whereas the original value from the TIFF mostly lies below or equals the one of the printed dogbones. Looking at the absolute numbers, the smallest absolute error of the developed simulation tool in comparison to the printed part can be found regarding the dimension “width_max_” (0.01 mm), while the smallest relative error occurs at the dimension “length” (0.07%). Concerning the highest error, both errors, the absolute (0.09 mm) as well as the relative (2.25%), are at the narrow middle part (width_min_).

**Figure 20:**
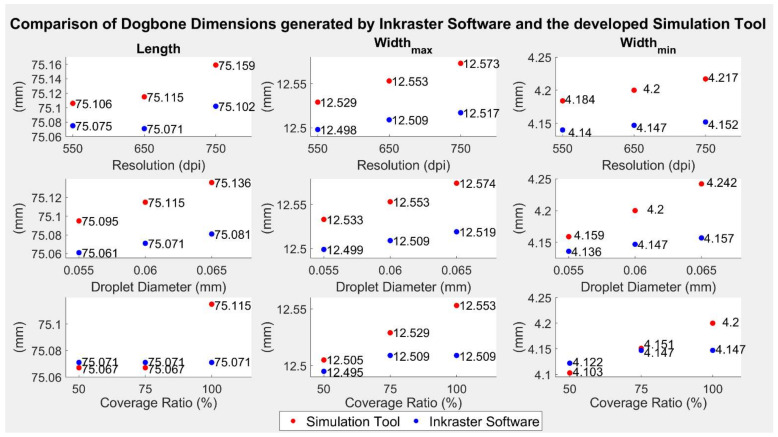
Results of the validation using the “Inkraster” software.

To further test the input parameter values, nine different sets of parameters were input in the developed simulation tool as well as in the software “Inkraster”. Then, again, the three dimensions were ascertained in the simulation tool and for the “Inkraster” output with the help of the gerber file viewer “GerberLogix” and its manual measurement tool. Hence, small inaccuracies might be found for the “Inkraster” values. The results are illustrated in Figure 20.

Generally, it can be seen that the trends within the figures were mostly similar, meaning that if the values simulated with the software “Inkraster” rose, the values simulated with the developed tool also rose. Furthermore, the deviations between the two tools were always less than 0.1 mm, which equaled approximately one drop at equilibrium.

It can also be seen that the values obtained with the developed tool were, in the majority of cases, higher than the ones of the software “Inkraster”.

Regarding the vertical parameters, the “KIT logo” samples were printed and simulated. The heights of the samples printed with the droplet diameter (*d*_0_*)* with two values of 41.2 μm and 31.9 μm, obtained by applying a gray level of 1:3 and 1:5, are shown in Table 1. A positive/negative relative error indicates that the simulated height is larger/smaller than the measured height.

Looking at the results listed in Table 1, it is not difficult to see that the dimensional deviations between simulation results and 3D-printed ones vary between −11.34% and 8.28% in general, except for a high error of 27.56% found in the case of the low resolution of 360 dpi, 75% coverage percentage and a large droplet diameter. This high error can be attributed to an insufficient center-to-center droplet distance leading to inadequate overlap distance between dispensed droplets, and thus an uncontrolled variation in printed features. The average relative error for a large droplet diameter is 10.89% and for a small droplet diameter is 8.09%. However, it is worth stating that in the case of the small droplet diameter (*d*_0_) of 31.9 μm, the relative errors for the sample printed with 360 dpi, 75% and 100% coverage percentages were much larger than the other relative errors. In particular, it is not possible to precisely predict the height for low resolution, namely 360 dpi, with small droplet diameters. The equilibrium drop diameter in this case is smaller than the distance between adjacent pixels, which leads to an underfilled layer, and the underfilled sample is not taken into consideration by the simulation tools.

## 5. Discussion

In this section, the applicability of the developed simulation tool will be discussed. In the previous section, deviations between simulation results and 3D-printed parts were found. The reason for these differences could be that the time until the material was cured was not considered when implementing the simulation tool. Hence, it could be that the material was cured with the UV lamp before the droplets reached their equilibrium shape. This means that the contact angle would be higher, and therefore so would the drop height and eventually the layer height. How fast the droplets spread depends on other factors and the viscosity of the used ink.

**Figure 21:**
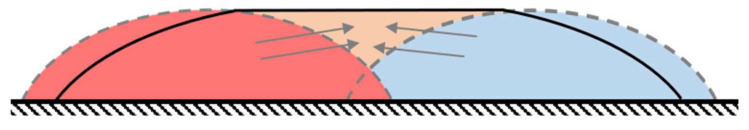
Cross-section of two coalescing drop lines and the resulting surface.

Moreover, in the simulation tool, only drop lines are created and not drop arrays. However, it could be assumed that when placing several drop lines next to each other that those would coalesce and tighten up in order to form a planar surface, as found by Zhou (see Figure 21). This would explain why the simulated horizontal dimensions were larger than the measured ones.

Another cause for the dimensions of the printed parts differing from the ones which were simulated might be that there is in fact a small loss of liquid/shrinkage due to evaporation when curing, resulting in the shrinkage of the parts. Likewise, the values obtained from the simulation tool were higher than those obtained from the software “Inkraster”. This could be put down to the fact that “Inkraster” does not take the coalescing and line formation of the drops into account, but only displays single drops with their diameters, whereas the developed tool uses the line width in case the drops connect. Since the line width *w* is, in this case, greater than the droplet diameter at equilibrium *d_eqm_*, the part dimensions will also be wider when applying a coverage percentage of 100%. Due to the fact that the line width *w* also incorporates the drop spacing *p*, it gets smaller with a decreasing coverage percentage, which might be the cause for the lower numbers of the developed simulation tool regarding a coverage percentage of 50% or 75%.

As “Inkraster” only places a drop onto every second pixel (50% coverage percentage), but still has the same diameter, this value does not decrease as much as the one of the developed tool, which takes the higher drop spacing into account.

Looking at the validation results, it can be said that the developed simulation tool produces values which are close to the measured dimensions of the printed parts and values produced by similar software programs. Nevertheless, there seem to be deviations, which might be reduced by including the curing time and a more precise simulation with drop arrays. However, some difference between the values will always be found due to printer inaccuracies and tolerances, as well as different software and application principles. Therefore, one can say that the simulation tool is close to the dimensions encountered in reality and applicable to the 3D inkjet printing process, especially to obtain a first impression about the effects of the parameter settings which will be applied to a printing process.

### Comparison of Results to Related Studies

In this section, the findings of this work will be placed in scientific context and compared to the previously identified results of other studies.

This work aims to fill the gap on the uninvestigated effects of TIFF input on the 3D inkjet process by developing a simulation tool with which the behavior of ink droplets on the substrate can be modelled and simulated, and using that tool to predict the influence of process parameters on the dimensional accuracy of printed parts.

Placing the findings of this study into a scientific context consisting of the studies and works presented in the Introduction, it can be said that, to the best of the authors’ knowledge, no similar work has been reported. Nevertheless, studies aiming to simulate and optimize the 3D inkjet printing process with regard to different aspects have been introduced. This makes it difficult to actually compare obtained results, but the differences and similarities of studies and results will still be discussed, in addition to how the results of this work could be of benefit to other studies.

In contrast to the tool developed by Guo et al. [22,23,24], the one from this work is applied before the printing process is started, aiming to identify optimal process parameters beforehand. Additionally, there is no automatic feedback of the tool to the system, meaning the user needs to detect errors himself and find a way to reduce and optimize the applied parameters. However, the developed simulation tool could help to train the developed algorithm of Guo et al. [24] to detect and classify errors and their causes faster.

Combining the prediction model of Salcedo et al. [27] and this simulation tool, drop patterns influencing the deformation or stress concentration of specific parts could be detected, and parts could therefore be tailored to their function. Additionally, the material classification system created by Zhou et al. [28] could be included in or combined with this tool, since it defines ink fluid properties such as surface tension and viscosity, which are also crucial for this simulation tool. Moreover, the general printability of materials could then be predicted, and the behavior of the material and decisions on which material to use for the desired part and required application could be facilitated.

A work rather similar to this one is the study of Wu et al. [29] which investigated the formation of droplets, i.e., the time before the drops reach the substrate. The developed simulation tool does not consider the formation of satellite drops but assumes that all drops reach the substrate in an ideal condition, meaning without satellite drops and without splattering. This, however, cannot be guaranteed and is only achievable by finetuning the print heads and nozzles exactly to the material properties, printing speed, and distance between nozzles and substrate. Hence, by including the findings of Wu’s et al. study [29], the tool could achieve greater accuracy and be closer to real printing conditions.

Looking at the comparisons made above, it can be said that this work is a further step to better understand the 3D inkjet printing process. It covers the need for investigating the behavior of droplets on a substrate before the process is started.

## 6. Optimization-Oriented Simulation of the Inkjet Process

**Figure 22:**
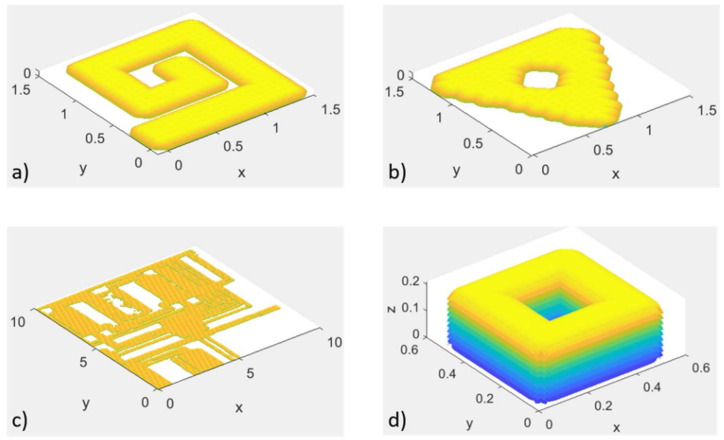
Simulation of different shapes with one layer in (**a**–**c**) and a simulation of multiple layers shape in (**d**).

After experimentally validating the proposed simulation tool, different simulation trials under different conditions were performed. In Figure 22, simulations of different shapes and parts are illustrated. Pictures (a) and (b) visualize a single simulated layer. In picture (b), the effects of the pixels are clearly detectable at the edges of the triangle and in the shape of the circle in the middle. However, pictures (c) and (d) show a more practical usage, with picture (c) displaying the etching of a circuit board and picture (d) a basic 3D part. In the latter, the layer structure and stacking are also visible.

**Figure 23:**
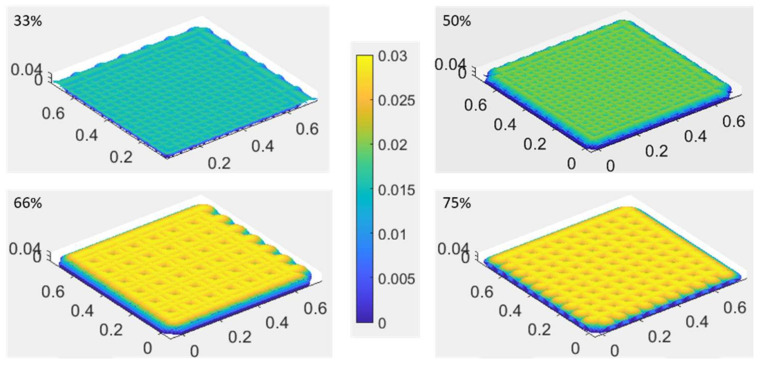
Illustration of the simulation of a TIFF with different coverage percentages.

To illustrate the effect of different coverage percentages, a single layer of a simple square shape was modelled (see Figure 23). The effect on the drop spreading and hence the ink density is noticeable, as is the effect on the layer height. Generally, one can say that, with a rising coverage percentage, the layer height also rises, although the layer height of the square simulated with a coverage percentage of 66% (0.026 mm) was slightly higher than the one simulated with 75% (0.025 mm).

By using the simulation tool, the coverage of the TIFF pixels by the liquid ink can be controlled and, if below 100%, readjusted by changing the input parameters of resolution or coverage percentage, since the drop diameter is mostly predefined through the printhead and its nozzles. To obtain fully functional and solid parts, in the majority of all cases, a coverage of 100% is required. To accomplish that, the following conditions need to be fulfilled:

1. $d_{eqm}\geq l_{px}*\;\frac{1}{Coverage\;percentage\;}$
2. $d_{eqm}\geq w_{px}*\;\frac{1}{Coverage\;percentage\;}$
3. $w_{x}\geq l_{px}*\;\frac{1}{Coverage\;percentage\;}$
4. $w_{y}\geq w_{px}*\;\frac{1}{Coverage\;percentage\;}$

Where conditions 1 and 2 indicate that single black pixels are completely covered by single drops and conditions 3 and 4 ensure that drop lines spread over the whole pixel, meaning that parallel drop lines need to connect to the ones next to them.

The functioning of the simulation tool in its current version helps to understand how drops spread and merge, and what influence the resolution and coverage percentage have onto the part to be built. However, to make it even more accurate and tailor it to the used printer and process, several functions should be implemented and added in the future.

By further investigating the coalescence of drops and how drop arrays form and behave, the modelling of the drops on the substrate could be improved, especially through identifying, defining and establishing mathematical and physical models through which it is possible to plot the drop arrays in detail. The current illustration using drop lines in the x- and y-directions seems to be quite effective and close to reality. However, with further studies and investigations, the accuracy and applicability of the developed tool could be optimized.

In addition, as already earlier mentioned in this work, the time until curing is an essential factor, as well as the curing itself. Therefore, a study on the spreading time of drops, also considering the viscosity of the ink and the effect of the UV light intensity, should be conducted and, using these results, the simulation tool improved and its value enhanced. In line with this, the ejection speed and the speed of the moving printhead, which determine how and in which angle the droplets fall and whether satellite drops occur, could be investigated. In future, it should be taken into account that, after the first layer is applied, the base surface for the next layer is not as smooth as the one of the substrate for the first layer.

As another topic for further research, the economic consequences and benefits of the developed tool could be looked at more closely, and the profits of the usage of the tool could be determined.

## 7. Conclusions

The aim of this study was to investigate the correlation between the properties of a TIFF—i.e., its resolution and pixel coverage percentage—and the size of droplets and their behavior on a substrate in the 3D inkjet printing process by creating and validating a simulation tool. To achieve this aim, first an introduction to 3D inkjet printing and the physics of droplets was given, and the main characteristics of a TIFF were explained. Then, the simulation tool was developed using MATLAB and tools and methods for the validation of the horizontal and vertical features were presented. In light of these steps, the main results of this work are:

- A summary of droplet behavior entailing theory and equations to model droplets/substrate interactions;
- A geometric-based approach to simulate drop coalescence on a substrate considering resolution and coverage percentage of the TIFF file and droplet diameter;
- A ready-to-use simulation tool which models the behavior of ink droplets in a multilayer 3D inkjet printing process;
- A validation of the aforementioned tool showing the general agreement of the simulations performed with the tool and the printed parts;
- An applicability evaluation highlighting aspects to consider and improve in future works.

In more detail, it can be concluded that the simulation tool helps to visualize the drop behavior on the substrate, as well as the effects of resolution, coverage percentage and drop diameter. Furthermore, by using the tool, possible errors and inaccuracies can be detected before the printing process is started, and hence avoided.

The results showed the general applicability of the tool and helped to identify the limitations and weaknesses of the tool, which can be corrected in the future through further implementation, following tests and validation approaches.

All in all, this work contributes to a better understanding of the 3D inkjet printing process and its process parameters and helps to improve the quality of printed parts, as well as the applicability of the process to different industry applications and fields.

## Figures and Tables

**Table 1:** Simulated and measured sample heights for different printing conditions.

	Simulated (mm)	Measured (mm)	Relative Error
Droplet diameter (*d*_0_) of 41.2 μm			
360 dpi with 75% coverage percentage	0.162	0.127	27.56%
360 dpi with 100% coverage percentage	0.156	0.167	−6.59%
720 dpi with 75% coverage percentage	0.493	0.535	−7.85%
720 dpi with 100% coverage percentage	0.681	0.725	−6.07%
1080 dpi with 75% coverage percentage	0.874	0.825	5.94%
1080 dpi with 100% coverage percentage	1.024	1.155	−11.34%
Droplet diameter (*d*_0_) of 31.9 μm			
720 dpi with 75% coverage percentage	0.569	0.532	6.95%
720 dpi with 100% coverage percentage	0.785	0.725	8.28%
1080 dpi with 75% coverage percentage	0.882	0.815	8.22%
1080 dpi with 100% coverage percentage	1.034	1.135	−8.90%

## Data Availability

The data presented in this study are available on request from the corresponding author.
